# Feature Optimization Method of Material Identification for Loose Particles Inside Sealed Relays

**DOI:** 10.3390/s22093566

**Published:** 2022-05-07

**Authors:** Zhigang Sun, Aiping Jiang, Guotao Wang, Min Zhang, Huizhen Yan

**Affiliations:** 1 Electronic Engineering College, Heilongjiang University, Harbin 150080, China; 2191313@s.hlju.edu.cn (Z.S.); 1988014@hlju.edu.cn (A.J.); 20170735@s.hlju.edu.cn (M.Z.); 2 Reliability Institute for Electric Apparatus and Electronics, Harbin Institute of Technology, Harbin 150001, China; 2191316@s.hlju.edu.cn

**Keywords:** sealed relays, material identification, missing value processing, standardization and normalization processing, feature selection, RF classifier

## Abstract

Existing material identification for loose particles inside sealed relays focuses on the selection and optimization of classification algorithms, which ignores the features in the material dataset. In this paper, we propose a feature optimization method of material identification for loose particles inside sealed relays. First, for the missing value problem, multiple methods were used to process the material dataset. By comparing the identification accuracy achieved by a Random-Forest-based classifier (RF classifier) on the different processed datasets, the optimal direct-discarding method was obtained. Second, for the uneven data distribution problem, multiple methods were used to process the material dataset. By comparing the achieved identification accuracy, the optimal min–max standardization method was obtained. Then, for the feature selection problem, an innovative multi-index–fusion feature selection method was designed, and its superiority was verified through several tests. Test results show that the identification accuracy achieved by RF classifier on the dataset was improved from 59.63% to 63.60%. Test results of ten material verification datasets show that the identification accuracies achieved by RF classifier were greatly improved, with an average improvement of 3.01%. This strongly promotes research progress in loose particle material identification and is an important supplement to existing loose particle detection research. This is also the highest loose particle material identification accuracy achieved to in aerospace engineering, which has important practical value for improving the reliability of aerospace systems. Theoretically, it can be applied to feature optimization in machine learning.

## 1. Introduction

As an important part of various pieces instruments, the reliability of sealed relays directly affects the reliable operation of aerospace equipment [1]. Among many reliability problems associated with sealed relays, the loose particle problem is the most important. The production process of sealed relays is relatively complex. In the production process, copper wires, solder particles, aluminum chips, wire skins, and other particles may be encapsulated inside the relays [2]. These substances generated inside or brought in from outside and destroy the original state of the relays are called loose particles [3]. Under overweight, weightless, or sudden shock conditions, loose particles inside sealed relays could move randomly [4]. They may strike internal contacts or components, causing inoperative phenomenon or abnormal operation of the sealed relay [5]. An invalid sealed relay could cause operational failure of aerospace equipment and lead to aerospace accidents, resulting in huge losses [6,7].

Particle impact noise detection (PIND) experiments can be used to effectively detect loose particles inside sealed relays [8,9,10] and screening for invalid products [11,12]. From the perspective of the entire sealed relay production process, such testing occurs during pre-delivery inspection at the end of the production process. During the inspection process, if it can be determined not only whether there are any loose particles inside the sealed relay but also the material constituting the loose particles, the production link that produced the loose particles can be traced. In this way, by further standardizing details and requirements of the corresponding production link, the loose particle problem can be effectively reduced. In recent years, the Loose Particle Detection Research (LPDR) group of the Institute of Electrical and Electronic Reliability, Harbin Institute of Technology, where the authors are located, has been devoted to the exploration of and research on loose particles, such as their materials, locations, and weights [13,14,15,16,17,18,19,20]. By combining signal processing technology and machine learning methods, the group aims to provide reference information for the inspection of loose particles in aerospace engineering.

One of the research directions of the authors is the material identification of loose particles inside sealed relays. On the basis of a sufficient literature review and experimental testing in the early stage, we propose to transform the loose particle material recognition problem into a multi-classification problem in machine learning. The theoretical basis for conducting research in this way is that if acoustic emission sensors were placed on the surface of a sealed relay, the sensors could collect the generated loose particle signals. Thus, if the materials of the loose particles were different, the loose particle signals generated by the collision would also be different. This would allow for analysis of the differences, which could be applied it to build a feature dataset and, ultimately, trained classifiers with different materials as labels. Specifically, we placed loose particles of different materials inside sealed relays and generated loose particle signals. The loose particle material dataset (material dataset) can be formed by extracting the pulse area, spectral centroid, and other features that could reflect the material information from the signals. Combined with the Random Forest algorithm, the classifier was trained on it, and a detailed description can be found in Section 2.

Following this idea, in previous research, we extracted fourteen time-domain and frequency-domain features from loose particle signals and built a material dataset. We trained the classifier on the material dataset based on Decision Tree and Random Forest and concluded that the Random-Forest-based classifier (RF classifier) achieved the best classification results, i.e., achieved the highest material identification accuracy. The RF classifier achieved the highest material identification accuracy on the loose particle dataset of 59.63%. It should be noted that in the early stages, we focused on improving the classification performance of the classifier in algorithm selection and parameter optimization, which ignored the material dataset, especially the features and data distribution in the dataset. In machine learning, high-quality datasets are often more important than good classification algorithms [21]. The features are the basis for constructing the dataset. Therefore, the better the feature optimization effect of the dataset, the higher the quality of the dataset, the better the classification performance of the trained classifier, and the higher the identification accuracy achieved.

In machine learning, work related to feature processing of datasets is known as feature engineering. Many scholars have carried out research on feature engineering and achieved good research results. For example, Ma et al. [22] proposed a feature selection method for a forest optimization algorithm based on contribution degree. The proposed method used a contribution degree policy embedded in the forest optimization algorithm. The goal of the contribution degree was to guide the search process of the forest optimization algorithm to select features according to high correlation and low redundancy among them. The algorithm was fully validated on datasets from the UCI repository. Combining adversarial-based learning and distributed techniques, Wu et al. [23] proposed a new hybrid binary particle swarm optimization method incorporating information gain theory. The converted information gain value was used as a weight coefficient to adaptively adjust the flight speed of the particles. A support vector machine (SVM) algorithm was applied to evaluate the performance of feature optimization from two perspectives, including user identification accuracy and feature reduction rate. Wang et al. [24] proposed an ensemble feature selection algorithm based on a multimodal optimization technique. Differential evolution based on a fitness Euclidean-distance ratio (FERDE) algorithm was utilized to search for multiple diverse feature subsets in the huge feature space. A set of diversity-based classifiers was constructed based on these subsets and ensembles to improve the final classification performance. Sreedharan et al. [25] adopted a feature extraction method based on scale-invariant feature transformation to extract features from face points. Later, a meta-heuristic called grey wolf optimization (GWO) was used to select the best features. Demir et al. [26] investigated missing data and redundant features and proposed a method including missing feature completion with statistical moments (means) and feature selection using novel group optimization methods. To select meaningful features, a feature selection method based on chaotic Darcy optimization was proposed, which selected the thirty-one most discriminative features of the complete HCC dataset. Aghdam et al. [27] proposed a novel feature selection method based on particle swarm optimization to improve the performance of text classification. Particle swarm optimization is inspired by the social behavior of fish flocking or bird flocking. The complexity of the proposed method is very low due to the application of a simple classifier. Yazdani et al. [28] proposed two feature selection methods by modifying the main operators of the biogeography-based optimization algorithm. The difference between these methods is the use of binary versus integer encoding. Simulations were performed on datasets with different feature dimensions and categories. Pourpanan et al. [29] combined incremental learning fuzzy min–max (FMM) neural networks with brain storm optimization (BSO) to take on feature selection and classification problems. Ten benchmark questions and a real-world case were used for testing and research to evaluate the effectiveness of the proposed FMM-BSO.

Based on summarizing the relevant research results, we chose to shift our research focus to the features in the material dataset and thus proposed a feature optimization method of material identification for loose particles inside sealed relays. First, the missing values in the material dataset were processed, which is summarized as the missing value processing stage. We used a variety of statistical methods to fill in the missing values and applied the RF classifier to make predictions on the processed dataset. Thus, multiple identification accuracies were obtained. We used the direct-discarding method to process the missing values and applied the RF classifier to make predictions. By comparing the identification accuracies achieved by the RF classifier on the datasets processed by statistical method with those achieved by the direct-discarding method, the best missing value processing method can be obtained. Second, different standardization and normalization processing methods were used to deal with the problem of large difference in numerical distribution between features in the dataset, which was summarized as the standardization and normalization processing stage. Specifically, we used z-score standardization, min–max standardization, and row normalization to process the material dataset and applied the RF classifier to make predictions. The optimal standardization and normalization processing method can be obtained by comparing the obtained identification accuracies. Then, we studied the feature selection method based on the “filtering method” and designed a multi-index-fusion feature selection method, which is summarized as the feature selection stage. We used the new feature selection method to select features that contribute considerably to the identification accuracy of the RF classifier on the dataset and further constructed a new material dataset. The RF classifier achieved the highest identification accuracy on this new dataset. With this, we had obtained the best missing value processing method, the best standardization and normalization processing method, and the multi-index-fusion feature selection method and completed the design of the feature optimization method proposed in this paper. Finally, we constructed several material verification datasets to test the above feature optimization method. The practicability and robustness of the proposed method were proven by comparing the identification accuracy achieved by the RF classifier on the verification datasets before and after optimization.

## 2. Related Works

### 2.1. Loose Particle Material Identification Experimental System

**Figure 1:**
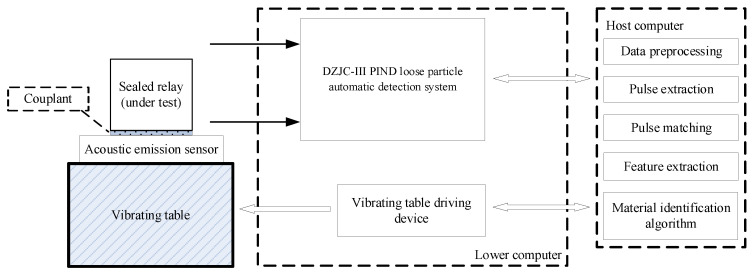
Block diagram of the loose particle material identification experimental system.

**Figure 2:**
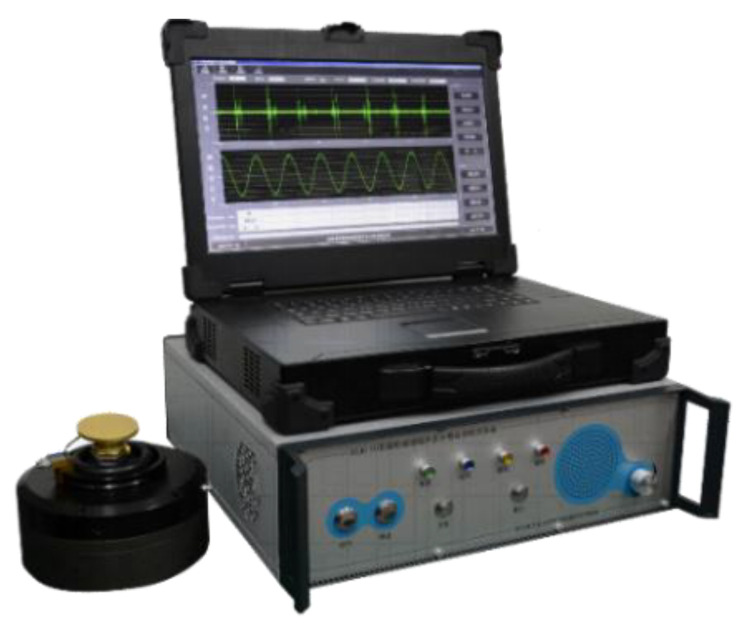
DZJC-III PIND loose particle automatic detection system.

We used the DZJC-III PIND loose particle automatic detection system (loose particle automatic system), which was independently designed and manufactured by the LPDR group, to build the loose particle material identification experimental system, as shown in Figure 1. The system consists of three parts. The first part is the PIND experimental platform, including the sealed relay (under test), vibrating table, vibrating table driving device, couplant, etc. It is used to stimulate the sealed relay so that the loose particles inside the relay collide with the inner wall of the relay and a loose particle signal is generated. The second part is the loose particle detection system, as shown in Figure 2. The acoustic emission sensor provided by the system is placed on the surface of the relay, which is used to capture the generated loose particle signal, and is implemented in the system for signal conditioning and synchronization. Finally, the digital signal data are saved on the host computer in “dat” format [30]. The third part is algorithm processing, mainly completed on the host computer. Based on the obtained signal data, steps including data preprocessing, pulse extraction, pulse matching, feature extraction and the material identification algorithm (machine learning classification algorithm) are completed.

### 2.2. Technology Implementation Process

Based on the proposed loose particle material identification experimental system, the implementation processes of loose particle material identification are as follows.

**Step 1:** Multiple sealed relay samples of the same type as the sealed relay (under test) were prepared in advance, and each sealed relay sample contain loose particles of only one type of material. Therefore, ***N*** sealed relay samples contain loose particles of ***N*** materials inside. We selected one of the sealed relay samples.

**Step 2:** The selected sealed relay sample was fixed to the PIND experimental platform, and mechanical excitation was applied to it by driving the vibrating table so that the loose particle inside the sample would be in a collision or sliding state.

**Step 3:** The loose particle signal collected by the acoustic emission sensor was sent to the loose particle detection system for conditioning and collection, and the collected signal data were sent to the host computer for storage.

**Step 4:** The signal data were processed, and multiple features that reflect the material information were extracted from the processed signal data to form the material data, i.e., the data representing the material of the loose particle inside the sealed relay sample selected in Step 1.

**Step 5:** The sealed relay sample selected in Step 1 was adjusted, in fact, it is to adjust the material of the loose particle contained inside the sealed relay. Step 1 to Step 4 were to obtain the data representing the new material. The material dataset representing different materials of loose particles was built by selecting the **1st** to ***N*th** sealed relay samples in order and repeating Step 1 to Step 4.

**Step 6:** Multiple classifiers based on different machine learning classification algorithms were trained on the material dataset, the prediction performance of each classifier was evaluated, and the best-performing classifier was obtained. Then the inherent parameters were optimized to achieve optimal performance.

**Step 7:** The sealed relay (under test) was fixed on the PIND experimental platform, and mechanical excitation was applied to cause the loose particle within to be in a collision or sliding state. Step 3 and Step 4 were repeated, the material data to be predicted (without labels) were compiled, and the optimal classifier was applied to make predictions. Then, the predicted loose particle material was obtained.

In our previous research, we used the optimal RF classifier mentioned above. At present, the highest identification accuracy achieved by the RF classifier on the existing material dataset is 59.63%.

### 2.3. Sealed Relay Samples

**Figure 3:**
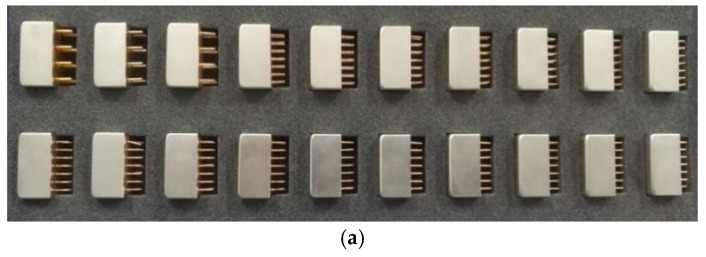

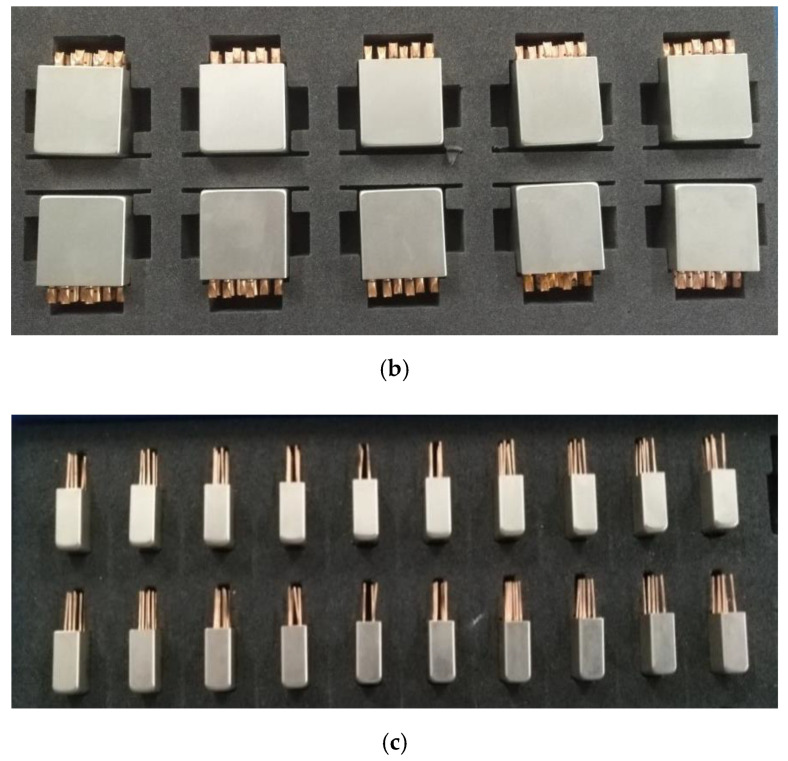
Sealed relay samples. (**a**) Sealed relay samples of model one; (**b**) sealed relay samples of model two; (**c**) sealed relay samples of model three.

Based on common loose particles found inside sealed relays, we chose copper wires, solder particles, aluminum particles, hot glue particles, wire coatings made of PVC material (PVC particles), wire coatings made of silica gel (silica gel particles)—a total of six kinds of materials—as the experimental objects. Considering the improvement in manufacturing sealed relays in recent years, a large number of test results show that the number of loose particles inside sealed relays was does not exceed three; therefore, our sealed relay sample contained one loose particle. The LPDR group coproduced, with Guizhou Space Appliance Co., Ltd., Guizhou, China, and obtained sealed relay samples containing loose particles of the abovementioned six materials. A model of the sealed relay samples, material information, and weight information about the loose particles are shown in Table 1, and the sealed relay samples are shown in Figure 3.

### 2.4. Experimental Conditions

Based on the obtained sealed relay samples containing loose particles of different materials, we used the loose particle detection system to conduct experiments. The experimental conditions were set in strict accordance with the Chinese GJB65B standard [31] and are shown in Table 2.

We needed to select the weights of the loose particles contained in the sealed relay samples according to the sensitivity range of the acoustic emission sensor. In previous research, we tested whether the loose particle signal generated by loose particles of different weights could be collected by an acoustic emission sensor and obtained the weight range of the loose particles of the six materials used in the present experiment. Specifically, the weight range of silica gel loose particles is 0.6 mg to 1.6 mg, and the weight range of loose particles of other materials is 0.02 mg to 0.2 mg. It can be seen form the Table 1 that the weights of the loose particles contained in the sealed relay samples were all within the detectable range.

### 2.5. Feature Description

The loose particle signal generated by collision or sliding is an acoustic emission signal in a broad sense. From the perspective of acoustic emission signal identification, loose particle material identification is based on the principle of “inferring the acoustic emission source from the acoustic signal features”. Therefore, we selected the time-domain and frequency-domain features from the loose particle signal according to the above principle. First, the vibration frequencies generated by the collision of loose particles of different materials are different, and frequency-domain features, such as spectral centroid and Mersenne Cepstral coefficient, can be selected. Second, because the hardness of loose particles of different materials is different, the collision duration and the degree of close-to-elastic collision are also different. Time-domain features, such as pulse length and the degree of symmetry between left and right, can be selected. Because the masses of loose particles of different materials are different, the energies of the loose particles when they collide are also different. Time-domain features, such as pulse area and energy density, can be selected. In addition to the influence of the materials, the loose particle signal is also affected by the experimental conditions. Under different impact accelerations, vibration frequencies, and vibration acceleration conditions, the energies obtained by the loose particles detection system through different excitations are also different. Therefore, the experimental conditions can also be used as the loose particle signal features. It can be seen from Table 2 that the impact acceleration and vibration acceleration are fixed, so only the vibration frequency can be used as the loose particle signal feature.

Finally, we decided to extract a total of fourteen features from the signal, which reflect the material information of the loose particle. The specific descriptions are shown in Table 3. It should be noted that the “symbolic representation” in the table represents the name of the features in the dataset. Therefore, each piece of data in the material dataset is of 1 × 15 specification. The first column of the data represented in the label also represents the specific material. The second to fifteenth columns of data represent the fourteen abovementioned features.

## 3. Methods

The research processes of the proposed feature optimization method are as follows. First, statistical methods [32,33,34], such as mean, median, Lagrange interpolation [35], and Newton interpolation [36], were used to fill in the missing values in the material dataset, and the direct-discarding method was used for processing. We applied the RF classifier to make predictions on the dataset processed by the abovementioned methods; on which dataset the RF classifier achieved the highest identification accuracy. Then, the optimal missing value processing method was obtained, and the processed material dataset was saved. Second, the differences in the numerical distribution of each column feature in the material dataset were analyzed. We used z-score standardization [37], min–max standardization [38], and row normalization [39] methods to process the dataset so as to ensure that all rows and columns in the dataset were treated equally by the RF classifier. Then, the optimal standardization and normalization processing method was obtained, and the processed material dataset was saved. Then, we studied the feature selection method based on “filtering method” [40,41,42] and used Pearson coefficient [43] and *p*-value [44] to select the features in the material dataset. On this basis, we designed a multi-index-fusion feature selection method, which can effectively select the features that contribute considerably to the identification accuracy of the RF classifier in the dataset and obtained the final material dataset with the best quality, on which the RF classifier also achieved the highest identification accuracy. This completed the design process of the proposed feature optimization method, which will be described in detail below.

### 3.1. Missing Value Processing

With the help of the loose particle material identification experimental system, we obtained the initial material dataset. The dataset contains 1,039,776 pieces of data. Among them, there are 175,416 data points with the label “0”, 169,943 data points with the label “1”, 177,936 data points with the label “2”, 168,796 data points with the label “3”, 173,105 data points with the label “4”, and 174,580 data points with the label “5”. It should be noted that labels “0” to “5” represent the six materials in Table 1. Affected by many factors, such as low energy of the loose particle signal and discontinuous signal collection, some missing values inevitably appeared during the establishment of the material dataset. Table 4 lists detailed information about the missing values in the initial material dataset.

According to complete data in the dataset, five statistical methods, including mean filling, median filling, mode filling, Lagrange interpolation filling, and Newton interpolation filling, were used to process the missing values, and five processed datasets were obtained. In addition, we used the direct-discarding method to discard the whole section of data where the missing values were located, retaining the data without missing values to obtain the sixth dataset.

On this basis, we applied the RF classifier to make predictions on the above six datasets. In order to obtain relatively accurate prediction results, the RF classifier made ten predictions on each dataset and obtained ten prediction accuracies. We took the mean value of ten prediction accuracies as the final achieved identification accuracy in order to reduce the random influence. Table 5 lists the prediction accuracies achieved by the classifier on the six tested datasets.

It can be seen from the table that the RF classifier achieved the highest identification accuracy (59.63%) on the material dataset processed by the direct-discarding method. This is the same missing value processing method that we used previously. Meanwhile, the RF classifier achieved a slightly lower identification accuracy on the dataset processed by other statistical methods. Therefore, for the material dataset, the direct-discarding method is the best missing value processing method. This also shows that if the dataset contains a small proportion of missing values, they should be discarded directly without worrying about losing part of the information; this will improve the overall quality of the dataset. After the missing value processing stage, we a material dataset that does not contain missing values. The material dataset contains a total of 1,038,667 valid data points, which can be used for standardization and normalization processing.

### 3.2. Standardization and Normalization Processing

We further analyzed the numerical distribution of the features within the material dataset and found that the data scales between the features of each column were quite different. For example, the value range of the spectral centroid was 0.9 to 1, whereas the value range of the Cepstral coefficient difference was 400 to 800, and the data scale difference between the two was more than 400 times. Therefore, we standardized and normalized the material dataset, aiming to align the data distribution of the row features or column features within the dataset [45] and ensure that all the features in the material dataset are equally treated by the RF classifier. We selected the three methods of z-score standardization, min–max standardization, and row normalization.

#### 3.2.1. z-Score Standardization

The main purpose of z-score standardization is to unify the data of different levels into the same level and measure the data by the calculated z-score value so as to ensure its comparability [46]. The mean value of the processed data is zero, and the standard deviation is one [47]. The calculation formula is:

(1) z=(x−μ)/σ

In the formula, z is the normalized value, x is the pre-normalized value, μ is the mean value of the column, and σ is the standard deviation of the column.

#### 3.2.2. Min–Max Standardization

Min–max standardization replaces the input value with the output result of the following formula [48]:

(2) $$m=\left(x-x_{\min}\right)/\left(x_{\max}-x_{\min}\right)$$

In the formula, m is the normalized value, x is the pre-normalized value, $x_{\min}$ is the minimum value of the column, and $x_{\max}$ is the maximum value of the column.

#### 3.2.3. Row Normalization

Row normalization refers to the normalization of each row of the dataset, which means that the vector length of each row is the same [49]. We consider each row to be a vector in space:

(3) $$x=\left(x_{1},x_{2},\ldots ,x_{n}\right)$$

The $L^{2}$ norm of the vector, x, is defined as [50]:

(4) $$\mathrm{norm}\left(x\right)=\sqrt{x_{1}^{2}+x_{2}^{2}+\cdots +x_{n}^{2}},\;\;n=14$$

It can be seen from Equation (4) that we used the $L^{2}$ norm [51]. Other types of functions can also be chosen, as long as each row vector is constrained by the same type of norm.

#### 3.2.4. Results Analysis

We used the above three methods to process the material dataset obtained in the missing value processing stage. Similarly, we applied the RF classifier to make ten predictions on the three processed datasets, taking the mean value of the ten classification accuracies so as to indirectly characterize the processing effects of the three methods. Table 6 lists the prediction accuracies achieved by the RF classifier on the three datasets.

It can be seen from the table that the RF classifier achieved the highest average identification accuracy of 63.51% on the dataset processed by min–max standardization, which was significantly improved compared to 59.63% accuracy before processing (in the missing value processing stage). In addition, we can also see that the identification accuracies achieved by the RF classifier on datasets processed by z-score standardization and row normalization were also significantly improved, although there are still some gaps compared with the former. Similarly, after standardization and normalization processing, we obtained a material dataset with relatively regular data distribution, which can be used for feature selection.

### 3.3. Feature Selection

In machine learning, the prediction performance of the classifier increases with an increase in the number of used features [52]. However, when the number of features is oversaturated, redundant features degrade the prediction performance of the classifier. Therefore, it is necessary to select features from the original dataset that contribute most to the prediction performance of the classifier. It is necessary to filter out features that contribute considerably to the identification accuracy of the RF classifier. Feature selection can eliminate irrelevant and redundant features, thereby reducing the number of features, reducing the training or running time, and improving identification accuracy [53]. We studied the existing feature selection methods and adopted methods based on Pearson correlation coefficient and *p*-value. We performed feature selection on the dataset after min–max standardization processing and analyzed the feature selection effects. Furthermore, we effectively combined the feature selection method based on Pearson correlation coefficient and *p*-value and proposed a new multi-index-fusion feature selection method. Therefore, the effective features that contribute considerably to the identification accuracy of the RF classifier were selected, and a high-quality material dataset was constructed. The new feature selection method effectively improved the prediction performance of the RF classifier on the material dataset and considerably improved the identification accuracy of the existing loose particle material identification.

#### 3.3.1. Feature Selection Method Based on Pearson Correlation Coefficient

Pearson correlation is also called product–difference correlation or product–moment correlation. The Pearson correlation coefficient can be used to measure the linear relationship between the features of each column in the material dataset [54]. The greater the absolute value of the Pearson correlation coefficient, i.e., the closer the correlation coefficient is to 1 or −1, the stronger the correlation between the two variables used in the calculation. The closer the correlation coefficient is to 0, the weaker the correlation between the two variables [55]. Assuming that there are two variables, X and Y, the Pearson correlation coefficient between the two variables can be calculated as follows [56]:

(5) $$\rho_{X,Y}=\frac{cov\left(X,Y\right)}{\sigma_{X}\sigma_{Y}}=\frac{E\left(XY\right)-E\left(X\right)E\left(Y\right)}{\sqrt{E\left(X^{2}\right)-E^{2}\left(X\right)}\sqrt{E\left(Y^{2}\right)-E^{2}\left(Y\right)}}$$

In the formula, E represents the calculation of mathematical expectation between two variables, and cov represents the calculation of covariance between two variables.

**Figure 4:**
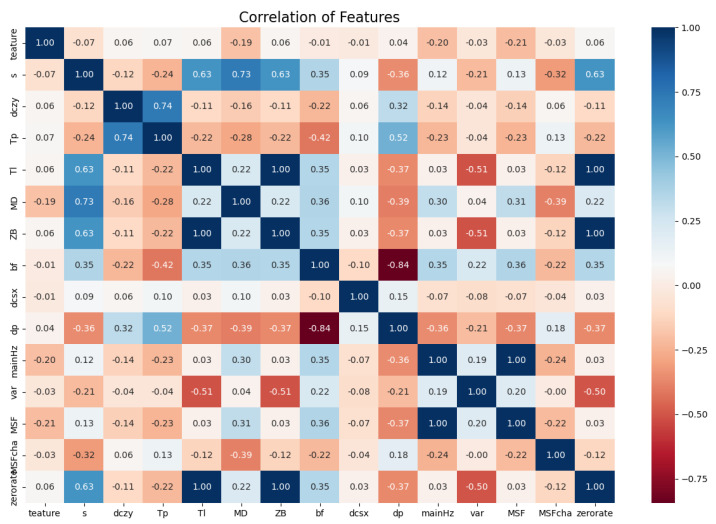
Heat map of the material dataset.

When using the feature selection method based on Pearson correlation coefficient on the material dataset, we treated labels as a fixed variable and other column features as another variable. Thus, Pearson correlation coefficients between each column feature and the label can be calculated. In this case, the closer the calculated correlation coefficient is to 1 or −1, the more important the column features used for calculation. Values of the calculated correlation coefficient closer to 0 indicate that the column features used for calculation are relatively less important. We used *Pandas* to calculate Pearson correlation coefficients between each column feature and the label in the material dataset and draw a heat map, as shown in Figure 4.

In the heatmap shown in Figure 4, the lighter the color, the weaker the correlation between the two features; the darker the color, the stronger the correlation between the two features. The diagonal area from the upper left corner to the lower right corner represents the correlation of the feature with itself, so the color is the darkest. Taking this diagonal area as the dividing line, the two obtained triangular areas are actually the same. They both express the correlation between features. The value in each square shown in the figure represents the calculated Pearson correlation coefficient between the two features on the abscissa and ordinate of the corresponding square. For example, the first row of squares in the figure represent the Pearson correlation coefficients between the label and itself or between the label and the fourteen features. It can be seen that the correlation between labels and individual features is weak.

Furthermore, by setting the threshold of the absolute value of the Pearson correlation coefficient to 0.1, we selected and retained the three features of energy density (***MD***), spectral centroid (***mainHz***), and Cepstral coefficient (***MSF***). We processed the dataset after min–max standardization processing and reserved only the column data corresponding to the three column features to form a new dataset. The RF classifier was used to make predictions, and the achieved average identification accuracy was 48.76%. Compared with the identification accuracy achieved by the RF classifier on the dataset after min–max standardization processing, the identification accuracy decreased significantly. This is because the feature selection method based on Pearson correlation coefficient selected a small number of features, so a considerable amount of material information contained in the original dataset was lost.

The Pearson correlation coefficient describes the linear correlation between components. It can also be found from Figure 4 that the linear correlation between labels and features in the material dataset is weak. Therefore, we further investigated the non-linear correlation between labels and features.

#### 3.3.2. Feature Selection Method Based on *p*-Value

Hypothesis testing, also known as statistical hypothesis testing involves first making a certain hypothesis and then collecting data by sampling to make statistical inferences about whether the hypothesis should be rejected or accepted [57]. In feature selection, the principle of hypothesis testing is “whether the feature has a relationship with the response variable”. Therefore, the null hypothesis in this paper is “whether the features in the material dataset have a relationship with labels”, i.e., the response variable is the label. It is necessary to test each feature and determine whether it has a significant relationship with the label. To some extent, the detection logic of the feature selection method based on Pearson correlation coefficient described above is the same. Specifically, if the correlation between a feature and the label is too weak, then the hypothesis that the “feature has no relationship with the label” is considered true. If a feature is sufficiently relevant to the label, then the hypothesis can be rejected, and the feature is considered to be related to the label. *p*-value is a common evaluation index in hypothesis testing. The *p*-value is a decimal between 0 and 1 that represents the probability that given data appear by chance under hypothesis testing. The lower *p*-value, the greater the probability of rejecting the null hypothesis [58]. That is, in the material dataset, the lower the *p*-value, the greater the probability that a given feature is related to the label and the more it should be retained.

Commonly used hypothesis testing methods are Z test, *t*-test, chi-square test, F test, etc. In this article, the we to use chose the *t*-test. A *t*-test uses the t-distribution theory to infer the probability of a difference so as to compare whether the difference between two means is significant. In this article, the material dataset was established, and the feature data of the dataset were known. From another point of view, the built material dataset only contained limited feature data that did not fully reflect the value and distribution of all feature data. Therefore, for such a normal distribution with a finite number of samples and an unknown population standard deviation, *t*-test is most appropriate. In addition, Z test is a hypothesis testing method based on information about the normal distribution, given the known population mean and variance. The chi-square test is used for categorical variables. The values of the feature data in this article were continuous unknown values, rather than discrete categories. The F test is a hypothesis testing method for a known statistical model based on variance information. Therefore, none of these three methods are suitable for this research. The results of hypothesis testing can be seen as a description of the non-linear relationship between labels and features in the material dataset. Therefore, by studying the hypothesis testing results, analysis of the non-linear relationship between labels and features can be completed.

In machine learning, the threshold for *p*-value is 0.05; i.e., features with a *p*-value less than 0.05 are worth preserving. Therefore, we calculated the *p*-value of each feature and set the screening threshold at 0.05 to filter out the unqualified features in the material dataset. Ultimately, we selected a total of fourteen features, which is the same number of features as previously used, indicating that the effect of using this method for feature selection is not great. It can be seen from the feature selection effect that the non-linear correlation between labels and features in the material dataset is strong. Therefore, we considered comprehensive analysis and utilization of the linear correlation and the non-linear correlation between labels and features.

#### 3.3.3. Multi-Index-Fusion Feature Selection Method

According to the feature selection results based on Pearson correlation coefficient, the number of selected features is too small to construct a dataset containing sufficient information. It shows that the conditions for feature selection were too strict, with few features qualifying. According to the feature selection results based on *p*-value, all features in the material dataset were selected. This indicates that the conditions used for feature selection were too broad to filter out poor performers. Judging from the linear correlation or non-linear correlation between labels and features in the material dataset, there is a strong non-linear correlation and a weaker linear correlation between the two. From the global point of view, an extreme bias towards a certain correlation leads to an unsatisfactory feature selection effect. Therefore, we need to comprehensively consider both correlations to come up with a feature selection method that combines both correlations so that they are in a balanced state.

Based on the analysis and summary of the feature selection effects of the above two methods, we attempted to effectively combine the Pearson correlation coefficient and *p*-value to design a new multi-index-fusion feature selection method. In this method, we no longer used a single evaluation index to evaluate and select features. Instead, two evaluation indices were used together to evaluate the features in the dataset, and the final evaluation results of features were obtained after comprehensive consideration of two evaluation results. According to the results, we selected the features with excellent performance to achieve the purpose of feature selection. The specific implementation steps of the proposed method are as follows:

**Step 1**: Equation (6) was used to calculate the absolute values of Pearson correlation coefficient between features and labels in the material dataset, which are expressed as$\;r_{i}\left(i=1,2,\ldots ,14\right)$. Among them, i is the feature number, which is in the same order as that listed in Table 3.

(6) $$r=|\frac{1}{n-1}\overset{n}{\underset{i=1}{\sum }}\left(\frac{X_{i}-\overset{¯}{X}}{\sigma X}\right)\left(\frac{Y_{i}-\overset{¯}{Y}}{\sigma Y}\right)|$$

In the formula, $\frac{X_{i}-\overset{¯}{X}}{\sigma X}$, $\overset{¯}{X}$, and σX are the standard fraction, mean, and standard deviation of label $X_{i}$, respectively; and $\frac{Y_{i}-\overset{¯}{Y}}{\sigma Y}$, $\overset{¯}{Y}$, and σY are the standard fraction, mean, and standard deviation of feature $Y_{i}$, respectively.

**Step 2**: Based on the obtained absolute values of the Pearson correlation coefficient of each feature and label in the dataset, $r_{i}$ was ranked from large to small according to the numerical value. In this way, we obtained the first ranking number through$\;r_{i}$, expressed as $\;N^{i}\left(i=1,2,\ldots ,14\right)$.

**Step 3**: In the above process, we used a single evaluation index (Pearson correlation coefficient) to rank fourteen features. In the next step, we used the second index (*p*-value) to evaluate fourteen features in the same way. Similarly, we calculated the *p*-value(s) of all features in the dataset and expressed them as$\;s_{i}\left(i=1,2,\ldots ,14\right)$. We ranked $s_{i}$ from small to large according to the numerical value. In this way, we obtained the second ranking number, expressed as $\;N^{s}\left(s=1,2,\ldots ,14\right)$.

So far, we used the second index (*p*-value) to rank the fourteen features. Finally, it was necessary to conduct a comprehensive analysis based on the two ranking results to achieve the final evaluation of the fourteen features.

**Step 4**: According to the same feature, we accumulated the two ranking results to obtain fourteen cumulative sums, which are expressed as $\;E_{i}\left(i=1,2,\ldots ,14\right)$. We ranked $E_{i}$ from small to large according to the numerical value. Finally, we obtained the comprehensive ranking number, which is expressed as $\;N^{e}\left(e=1,2,\ldots ,14\right)$.

It should be noted that when $N^{e}$ of multiple features is the same, we made the following supplementary rules: the lower the ranking of $N^{i}$, the lower the comprehensive ranking of $N^{e}$ we artificially set; that is, the higher the priority. For example, $N^{i}$ = 3 and $N^{s}$ = 5 obtained the same $N^{e}$ with $N^{i}$ = 6 and $N^{s}$ = 2. However, under the supplementary rules, because the former $N^{i}$ = 3, the latter $N^{i}$ = 6; thus, the comprehensive ranking, $N^{e}$, of $N^{i}$ = 3 and $\;N^{s}$ = 5 is lower than that of $N^{i}$ = 6 and $N^{s}$ = 2.

**Step 5:** The feature selection experience shows that when the number of selected features accounts for more than half of the features in the dataset, the prediction effect obtained by the classifier can be ideal based on the dataset built by these selected features. Therefore, we combined the grid search method and retained the top eight to top fourteen features by referring to the comprehensive ranking number, $\;N^{e}$, and formed seven datasets. We applied the RF classifier to make predictions on each dataset and achieved multiple identification accuracies. By comparing on which dataset the RF classifier achieved the highest identification accuracy, the combination of features used to construct that dataset is optimal. Then, the optimal feature selection result was obtained.

We applied the multi-index-fusion feature selection method to the material dataset after min–max standardization processing and obtained rankings of fourteen features at different feature selection stages. The specific description is shown in Table 7.

According to the comprehensive rankings in Table 7, we formed seven new datasets by referring to the combination of the top eight to top fourteen features and implemented them in the grid search method. We applied the RF classifier to make ten predictions on seven datasets and found that the RF classifier achieved the highest average identification accuracy on dataset formed by the following twelve features: pulse area (***s***), degree of symmetry between left and right (***dczy***), pulse rise proportion (***Tp***), duration (***Tl***), energy density (***MD***), pulse ratio (***ZB***), area ratio (***dp***), spectral centroid (***mainHz***), variance (***var***), Cepstral coefficient (***MSF***), Cepstral coefficient difference (***MSFcha***), and Zero crossing rate (***zerorate***). The highest identification accuracy was 64.46%. The multi-index-fusion feature selection method retained twelve columns of feature data in the material dataset; 1×13 columns were formed, which is less than the original 1×15 columns, and the achieved identification accuracy was significantly improved compared with that before selection.

Table 8 lists the feature selection effects of feature selection methods based on Pearson correlation coefficient, *p*-value, and the multi-index-fusion feature selection method. It can be seen from the table that compared with the identification accuracy of 63.51% achieved by the RF classifier on the material dataset containing fourteen features, the RF classifier achieved an identification accuracy of 64.46% on the material dataset that contained twelve features after feature selection. Despite the reduction in two columns of feature data in the material dataset, the identification accuracy achieved by the RF classifier was improved by 0.95%. Similarly, we also found that compared to using a single evaluation index, the multi-index-fusion feature selection method organically combined the two evaluation indices and achieved an average identification accuracy higher than either of them. In other words, the average identification accuracy of the multi-index-fusion feature selection method is higher than that of the traditional feature selection based on the “filtering method”. This shows the superiority of the new feature selection method on the material dataset.

The proposed feature optimization method of material identification for loose particles inside sealed relays achieved the highest identification accuracy of 64.46% on the material dataset, which is significantly improved compared with 59.63% obtained in our previous study. Table 9 lists the identification accuracies achieved by the RF classifier in the missing value processing stage, standardization and normalization processing stage, and feature selection stage.

### 3.4. General Procedure

We summarized the above-mentioned research process and obtained the general procedures of the feature optimization method of material identification for loose particles inside sealed relays:

**Step 1**: Missing value processing stage. For the missing values in the material dataset, the direct-discarding method was used for processing.

**Step 2**: Standardization and normalization stage. In order to solve the problem of irregular data distribution in the material dataset, the min–max standardization method was used for processing.

**Step 3**: Feature selection stage. In order to improve the prediction performance of the RF classifier, the multi-index-fusion feature selection method was used to retain the features with a large contribution to the prediction performance of the RF classifier.

According above description, the overall quality of the processed material dataset was significantly improved compared with that before. This concludes the description of the general procedures of the proposed feature optimization method.

## 4. Verification and Analysis

In the previous description, we carried out missing value processing, standardization and normalization processing, and feature selection processing for the material dataset and obtained the optimal processing method at each stage. In the missing value processing stage, the direct-discarding method achieved the best performance. In the standardization and normalization stage, the min–max standardization method achieved the best performance. In the feature selection stage, the designed multi-index-fusion feature selection method achieved the best performance. In the “verification and analysis” stage, we prepared new sealed relay samples, used the loose particle material identification experimental system to conduct experiments, and built a material verification dataset. We tested the obtained optimal feature optimization methods of each stage on the material verification dataset, thus proving the robustness of the processed feature optimization method.

### 4.1. Preparation Works

#### 4.1.1. Material Verification Dataset

In order to verify the testing effect of the proposed feature optimization method of material identification for loose particles inside sealed relays in real-world application scenarios, we prepared new sealed relay samples. Following step 7 in the technology implementation process in Section 2.2 and with the help of the loose particle material identification experimental system, we obtained the verification steps in the “verification and analysis” stage. This is also an extension of Step 7 in the technology implementation process in Section 2.2.

**Step 1:** We fixed the sealed relay sample on the PIND experimental platform and applied mechanical excitation to it by driving the vibration table so that the loose particle inside the sealed relay sample was in a collision or sliding state.

**Step 2:** The generated loose particle signal was collected by the acoustic emission sensor provided by the loose particle detection system and sent to the detection system for signal conditioning and synchronization, and the collected signal data were sent to the host computer for storage.

**Step 3:** We processed the signal data and extract fourteen features that considerably contribute to the identification accuracy of the RF classifier from the processed signal data to obtain multiple pieces of data.

**Step 4:** We adjusted the sealed relay sample selected in step 1 and repeated steps 1 to 3 to obtain data representing the new material. By sequentially selecting the sealed relay samples, a material verification dataset representing loose particles of different materials can eventually be built.

**Step 5:** We followed the general procedures in Section 3.4 and performed feature optimization on the material verification dataset so as to obtain a high-quality material verification dataset.

**Step 6:** We applied the RF classifier to make predictions on the material verification dataset, obtained the predicted label of each data point in the dataset, and completed the prediction of the material of loose particles.

Following steps 1 to 4 and through a large number of balanced experiments, we built a material verification dataset representing the loose particles of six materials, the specific descriptions of which are shown in Table 10.

#### 4.1.2. Performance Evaluation Index

In order to evaluate the effect of the proposed feature optimization method, it is necessary to apply the RF classifier to make predictions on the processed material dataset and indirectly indicate the processing effect of the feature optimization method by measuring the obtained identification accuracy. Therefore, we selected the identification accuracy as the evaluation index to measure the prediction performance of the RF classifier.

Suppose that the dataset is$\;D=\left\{\left(x^{\left(1\right)},y^{\left(1\right)}\right),\ldots ,\left(x^{\left(N\right)},y^{\left(N\right)}\right)\right\}$, $y^{\left(i\right)}\in \left\{0,1,\ldots ,5\right\}$ is the ground truth corresponding to the feature data $\;x^{\left(i\right)}$, and $f^{\left(i\right)}\in \left\{0,1,\ldots ,5\right\}$ is the label predicted by the RF classifier. The identification accuracy achieved by the RF classifier can be expressed as the ratio of the number of data points correctly predicted to the total number of data points [59,60]. The formula is as follows:

(7) $$A=\frac{1}{N}\overset{N}{\underset{n=1}{\sum }}I\left(f^{\left(i\right)}=y^{\left(i\right)}\right)$$

In the formula, I is the indicator function; when $f^{\left(i\right)}=y^{\left(i\right)}$,$\;I\left(f^{\left(i\right)}=y^{\left(i\right)}\right)=1$.

### 4.2. Analysis of Feature Optimization Effects

Following Step 5 of the verification steps in Section 4.1.1, we first processed missing values in the material verification dataset. That is, the missing values in the dataset were directly discarded, and the RF classifier was used to make predictions on the processed material verification dataset, with an achieved identification accuracy of 67.53%. Next, min–max standardization processing was performed on the material verification dataset. We also applied the RF classifier to make predictions on the processed dataset, with an achieved identification accuracy of 69.88%. Finally, according to the results of the multi-index-fusion feature selection method, we retained the corresponding twelve columns of feature data in the dataset to form a new material verification dataset. We applied the RF classifier to make predictions, with a final achieved identification accuracy of 70.64%. Table 11 lists the identification accuracies achieved by the RF classifier on the material verification dataset after missing value processing, standardization and normalization processing, and feature selection. It should be noted that the above achieved identification accuracy is the average value of the prediction accuracy obtained by ten predictions so as to reduce the influence of random errors.

It can be seen from the table that the RF classifier achieved satisfactory identification accuracy on the material verification dataset processed in each stage, and the achieved identification accuracy in each stage improved significantly compared to the previous stage. This is consistent with the improvement trend of identification accuracy achieved by the RF classifier on the processed material dataset in each stage, which is shown in Table 9. This clearly demonstrates the practicability and robustness of the proposed feature optimization method of material identification for loose particles inside sealed relays.

Following the same verification steps, we once again prepared multiple sealed relay samples and ultimately built ten material verification datasets. We followed step 5 of the verification steps in Section 4.1.1, processed the ten material verification datasets, and obtained the feature optimization effects shown in Table 11. “Before optimization” refers to the identification accuracy achieved by the RF classifier on the material verification dataset built in step 4 of the verification stage. “After optimization” refers to the identification accuracy achieved by the RF classifier on the material verification dataset processed in step 5 of the verification stage. It should be noted that if there are missing values in the material verification dataset, the RF classifier cannot be trained on that dataset. Therefore, the identification accuracy of “before optimization” in Table 12 refers to the identification accuracy achieved by the RF classifier on the dataset after the missing value processing.

It can be seen from the table that the proposed feature optimization method not only achieved an obvious optimization effect on the material dataset but also achieved satisfactory identification accuracies on the ten material verification datasets, which effectively proves the practicability and robustness of the method in engineering applications. It can be concluded that the average improvement of identification accuracy achieved by the RF classifier was 3.01%. In the feature selection stage, we also used the feature selection methods based on Pearson correlation coefficient and *p*-value. It was found that most of the identification accuracies achieved by the RF classifier on the material verification datasets processed by these two methods were decreased, a small part was basically unchanged, and only one was slightly improved. This effectively illustrates the superiority of the multi-index-fusion feature selection method proposed in this paper.

## 5. Discussion

The problem of loose particles is an important factor that affects the reliable operation of sealed relays. With further research on loose particle detection technology, this problem has attracted increasing attention from scholars. Based on our previous research on material identification, in this paper, we transferred our research focus from the selection and optimization of classification algorithms to feature optimization of the material dataset and designed a complete set of feature optimization methods, including directly discarding missing values, performing min–max standardization processing, and applying the multi-index-fusion feature selection method. The proposed feature optimization method achieved considerable effects on both the material dataset and ten material verification datasets, and its feasibility was verified. The contributions of this paper can be summarized as follows:

(1) From another perspective, we considered how to improve the identification accuracy of existing loose particle material identification methods and transferred our research focus from the selection and optimization of classification algorithms to the optimization of the internal features in the material dataset.
(2) Methods in feature engineering were applied to conduct preliminary optimization processing on the material dataset, including missing value processing, standardization, and normalization processing. This effectively ensured a complete dataset and that the numerical distribution of all column features in the dataset had an approximately equal scale.
(3) The feature selection methods based on the “filtering method” were studied, and the feature selection effects of the feature selection methods based on Pearson correlation coefficient and *p*-value were analyzed. Accordingly, a multi-index-fusion feature selection method was designed. Thus, the features that contribute considerably to the prediction performance of the RF classifier were selected, and a new high-quality material dataset was constructed.
(4) The feature optimization method proposed in this paper was tested many times on validated datasets. Test results show that the RF classifier achieved the highest identification accuracy on the material dataset that processed by the proposed feature optimization method in this field. It has important reference value to trace the production process of loose particles in the manufacturing process of sealed relays. This method is an important supplement to existing loose particle detection technology and has extremely high application value for improving the reliability of aerospace system. In theory, it can be extended to the design of feature optimization methods for other datasets in machine learning.

It should be noted that the material dataset shown in Table 4 and the material verification dataset shown in Table 10 were both artificially controlled to maintain the data balance of each label in the dataset. However, in real-world applications, due to the differences among the materials, the energy and duration of the loose particle signals generated by the collision would be different, inevitability introducing the data imbalance problem. After conducting the same number of PIND experiments, the amount of data extracted from the loose particle signal generated by loose particles of some materials was about two times greater than that generated by loose particles of other materials. With this in mind, the first processing method the preferred method, i.e., artificially increasing the number of experiments of loose particles that formed less data. In combination with the LR-SMOTE algorithm put forward by Liang et al. [14] of the LPDR group, we will study the possible imbalance data problem from the algorithm level in the future.

It is also worth noting that the proposed multi-index-fusion feature selection method directly summed the ranking numbers based on Pearson correlation coefficient and *p*-value and then ranked according to the cumulative sum. In this process, we treated the evaluation results based on Pearson correlation coefficient and *p*-value equally. In the future, before directly accumulating the ranking numbers of the two, we will consider introducing the weight coefficient. In other words, we will multiply the two rankings by the weight coefficient and the opposite of the weight coefficient, respectively, and add the weighted rankings; then, a new ranking is carried out according to the new cumulative sum. In this way, by adjusting the weight coefficient, we can set the bias to the evaluation results based on Pearson correlation coefficient or *p*-value, and to some extent, we can avoid the situation of repeated comprehensive ranking numbers. For example, if$\;N^{i}$ = 3, $N^{s}$ = 5, and $N^{i}$ = 6, $N^{s}$ = 2 has the same $N^{e}$ = 8, and add the following weight coefficient regulations: $N^{i}$ multiplied by 0.8 weight coefficient, $N^{s}$ multiplied by 0.2 weight coefficient; then, the new $N^{e}$ of $N^{i}\;$ = 3, $N^{s}$ = 5 is 3.4, and the new $N^{e}$ of $N^{i}$ = 6, and $N^{s}$ = 2 is 5.2. It is obvious that the weighted comprehensive ranking number of $N^{i}$ = 3 and $N^{s}$ = 5 is smaller, so it will be selected first. It should be noted that weight settings can be adjusted according to actual conditions.

The most considerable innovation presented in this paper is the shift of research focus from the selection and optimization of the classification algorithms to the features within the material dataset. The root of the impact on the prediction performance of the RF classifier lies in the loose particle signal. Therefore, a more complete statement can be made as follows: the collection of high-quality loose particle signals is crucial to the preliminarily constructed material dataset, the material dataset after feature optimization is crucial to the preliminarily trained RF classifier, and the parameter-optimized RF classifier is crucial to the loose particle material identification accuracy. Therefore, in future research, it is necessary to study how to collect high-quality loose particle signals. On the one hand, it is necessary to study the selection and layout of acoustic emission sensors in order to collects loose particle signal with as much energy as possible. On the other hand, it is necessary to study the excitation conditions of loose particles, that is, to set the best matching excitation conditions for loose particles of different materials and different weights.

## 6. Conclusions

In our early research on loose particle material identification, we focused on the applicable machine learning classification algorithm and parameter optimization. In this paper, we chose to transfer our research focus to the features within the material dataset.

First, we compared the processing effects of mean filling, median filling, mode filling, Lagrange interpolation filling, Newton interpolation filling, and the direct-discarding method, which completed the tasks of the missing value processing stage and obtained the optimal direct-discarding method.

Second, we compared the processing effects of z-score standardization, min–max standardization, and row normalization, which completed the tasks of the standardization and normalization processing stage, obtaining the optimal min–max standardization processing method.

Then, we studied the feature selection methods based on Pearson correlation coefficient and *p*-value and combined the two to further propose a multi-index-fusion feature selection method, which completed the task of the feature selection stage. The feasibility and superiority of the proposed feature selection method were proven by several tests.

Finally, we applied the complete feature optimization route of “directly discarding missing values, performing min–max standardization processing, and applying the multi-index-fusion feature selection method” to the test of ten material verification datasets, which fully verified the practicability and robustness of the proposed feature optimization method. The test results of the material dataset show that the identification accuracy achieved by the RF classifier on the dataset before and after feature optimization was improved from 59.63% to 63.60%. The test results of ten material verification datasets show that the identification accuracy achieved by the RF classifier on the datasets before and after feature optimization was greatly improved, with an average increase in identification accuracy of 3.01%.

The test results show that the proposed feature optimization method of material identification for loose particles inside sealed relays has a significant effect on improving the prediction performance of the RF classifier, and the identification accuracies achieved on the dataset before and after optimization were significantly improved. Moreover, the proposed method performed well in multiple verification tests, which clearly proved its practicability and robustness. It is worth noting that the highest identification accuracy achieved by the RF classifier is also the highest identification accuracy achieved by loose particle material identification in the loose particle detection field to date.

## Figures and Tables

**Table 1:** Model of the sealed relay samples, material information, and weight information about the contained loose particles.

Model One		Model Two		Model Three	
Material	Weight	Material	Weight	Material	Weight
Copper wires	0.04 mg	Solder particles	0.03 mg	Aluminum particles	0.03 mg
	0.08 mg		0.07 mg		0.06 mg
	0.11 mg		0.11 mg		0.10 mg
	0.15 mg		0.15 mg		0.15 mg
	0.19 mg		0.19 mg		0.19 mg
Hot glue particles	0.03 mg	PVC particles	0.03 mg	Silica gel particles	0.6 mg
	0.08 mg		0.07 mg		0.9 mg
	0.12 mg		0.11 mg		1.2 mg
	0.15 mg		0.15 mg		1.4 mg
	0.16 mg		0.19 mg		1.6 mg

**Table 2:** Experimental conditions specified according to the Chinese GJB65B standard.

Impact Acceleration	Vibration Frequency	Vibration Acceleration
200 g	27 Hz	5 g
200 g	40 Hz	5 g
200 g	100 Hz	5 g

**Table 3:** Detailed description of loose particle features.

Feature Name	Feature Description	Symbolic Representation
Pulse area	Area of the pulse signal.	***s***
Degree of symmetry between left and right	Initiation process of the signal characterized by the symmetry angle.	***Dczy***
Characterize the onset speed of the signal from the perspective of the pulse rising speed	Initiation velocity of the signal characterized Pulse Rise Percentage.	***Tp***
Duration	Difference between the start and the end time.	***Tl***
Energy density	Measure of the distribution characteristics of the signal energy.	***MD***
Pulse ratio	Ratio of pulse duration to pulse length.	***ZB***
Crest factor	Extreme degree of the peak value in the waveform.	***bf***
Degree of symmetry between upper and lower	Ratio of rise time to fall time.	***dcsx***
Area ratio	Area ratio of the spectrum.	***dp***
Zero-crossing rate	Value of the sign change of a section of signal; its magnitude is related to the frequency of the signal.	***zerorate***
Variance	Used to calculate the difference between each variable and the overall mean.	***var***
Spectral centroid	Used to describe the spectral distribution and characterize the frequency of the loose particle signal.	***mainHz***
Cepstral coefficient	Obtained from the square root of the mean square frequency; used to describe the energy spectrum.	***MSF***
Cepstral coefficient difference	Obtained from the square root of the frequency variance; also used to describe the energy spectrum.	***MSFcha***

**Table 4:** Description of missing values in the material dataset.

Label	Total Number of Data Points	Number of Missing Values
0	175,416	231
1	169,943	243
2	177,936	186
3	168,796	84
4	173,105	208
5	174,580	157

**Table 5:** Prediction accuracies achieved by the RF classifier on six datasets.

	Method	Mean/%	Median/%	Mode/%	Lagrange Interpolation/%	Newton Interpolation/%	Direct Discarding/%
Number							
1		57.92	58.02	57.90	59.45	59.32	59.83
2		58.05	57.85	57.89	59.51	59.36	59.06
3		58.01	57.88	57.84	59.43	59.29	59.75
4		57.92	58.01	57.86	59.46	59.51	59.99
5		57.96	57.87	57.83	59.45	59.42	59.37
6		58.10	57.89	57.91	59.46	59.36	59.64
7		58.05	57.91	57.82	59.50	59.47	59.52
8		57.99	57.93	57.89	59.51	59.41	59.70
9		58.12	57.89	57.82	59.47	59.37	59.94
10		58.08	57.85	57.84	59.46	59.49	59.50
Mean value		58.02	57.91	57.86	59.47	59.40	59.63

**Table 6:** Prediction accuracies achieved by the RF classifier on the three datasets.

	Method	z-Score Standardization/%	Min–Max Standardization/%	Row Normalization/%
Number				
1		63.86	63.96	63.00
2		63.95	63.99	62.89
3		63.27	63.40	63.01
4		63.37	63.31	62.94
5		63.45	63.38	63.01
6		63.31	63.41	63.01
7		63.34	63.27	63.17
8		63.42	63.51	62.93
9		63.40	63.41	62.97
10		63.40	63.41	63.08
Mean value		63.48	63.51	63.00

**Table 7:** Feature selection effects of the material dataset.

	Stage	Absolute Value of Pcc ^1^	Ranking Number	*p*-Value	Ranking	Cumulative Sum of Rankings	Comprehensive Ranking
Feature							
***s***		−0.0701	4	0	1	5	4
***dczy***		0.0574	9	0	1	10	9
***Tp***		0.0684	5	0	1	6	5
***Tl***		0.0636	7	0	1	8	7
***MD***		−0.1945	3	0	1	4	3
***ZB***		0.0636	7	0	1	8	7
***bf***		−0.0062	14	4.325047 × 10^−7^	14	28	14
***dcsx***		−0.0137	13	1.521347 × 10^−28^	13	26	13
***dp***		0.0392	10	1.666064 × 10^−221^	10	20	10
***mainHz***		−0.2025	2	0	1	3	2
***var***		−0.0317	12	6.039703 × 10^−145^	12	24	12
***MSF***		−0.2077	1	0	1	2	1
***MSFcha***		−0.0331	11	2.512781 × 10^−158^	11	22	11
***zerorate***		0.0641	6	0	1	7	6

^1^ Pcc: Pearson correlation coefficient.

**Table 8:** Feature selection effects of different feature selection methods.

Method	Before Feature Selection/%	After Feature Selection/%	Increase/%
Pearson correlation coefficient	63.51	48.79	−14.72
*p*-value	63.51	63.51	0
Multi-index-fusion	63.51	64.46	0.95

**Table 9:** Identification effects in different processing stages.

Stage	Identification Accuracy/%
Missing value processing	59.63
Standardization and normalization	63.51
Feature selection	63.60

**Table 10:** Detailed description of the material verification dataset.

Label	Total Number of Data Points	Label	Total Number of Data Points
0	9994	3	10,006
1	10,021	4	10,105
2	9987	5	10,018

**Table 11:** Identification effects in different verification stages.

Stage	Identification Accuracy/%
Missing value processing	67.53
Standardization and normalization	69.88
Feature selection	70.14

**Table 12:** Feature optimization effects achieved by the RF classifier on ten material verification datasets.

Number	Before Optimization/%	After Optimization/%	Increase/%
1	67.53	70.14	2.61
2	64.82	68.19	3.37
3	60.89	64.13	3.24
4	62.05	64.88	2.83
5	61.22	64.27	3.05
6	65.70	68.16	2.46
7	64.69	67.75	3.06
8	62.49	66.08	3.39
9	63.76	66.91	3.15
10	66.62	69.58	2.96

## Data Availability

Some or all data, models, or code that support the findings of this study are available from the corresponding author upon reasonable request.
